# Chemistry towards Biology

**DOI:** 10.3390/ijms24043998

**Published:** 2023-02-16

**Authors:** Milos Hricovini, Josef Jampilek

**Affiliations:** 1 Institute of Chemistry, Slovak Academy of Sciences, Dubravska cesta 9, 845 38 Bratislava, Slovakia; 2 Department of Analytical Chemistry, Faculty of Natural Sciences, Comenius University, Ilkovicova 6, 842 15 Bratislava, Slovakia; 3 Department of Chemical Biology, Faculty of Science, Palacky University Olomouc, Slechtitelu 27, 783 71 Olomouc, Czech Republic

Although it may not seem like it, chemical biology has existed for a long time from today’s perspective. One of the earliest occurrences of the term “chemical biology” is in the book “On Fermentation” by Alonzo E. Taylor (1871–1949), published in 1907 [1]. Additionally, John B. Leathes (1864–1956) used this term in 1930 in his contribution “The Birth of Chemical Biology, the Harveian Oration before the Royal College of Physicians” [2] (full text freely available at [3]). Today, it is no longer possible to trace who first used the term/phrase “chemical biology”, but it is clear that the concepts of chemical biology go back a long way [4]. A review by Morrison and Weiss [5] also offers a well-founded insight into the history of chemical biology.

Currently, chemical biology can be considered a bridge closely connecting chemistry and biology; the application of chemical techniques (analysis, synthesis, and computational chemistry) to the in vitro and in vivo studies of biological systems, where the study is based on the use of small molecules designed or identified on the basis of biochemical or cellular screening. These molecules are targeted to bind to biological structures, and the biological response is monitored [6]. Thus, chemical biology has become an important interdisciplinary field that is rooted in/seeks to link bioorganic, medicinal, and supramolecular chemistry with genetics, biochemistry, molecular biology, metabolic engineering, and pharmacology, using all known available analytical/bioanalytical methods or tools [7]. Thus, it attempts to explain the molecular nature of biological processes and interpret biological data/observations using various chemical principles.

In general, it is thus possible to define chemical biology as the study of molecular mechanisms of biological processes through targeted designed molecules.

In the shadow of the above definition, however, is the contribution of Hricovini et al. [8]. An evolutionary overview of molecular interactions and enzymatic activities in the yeast cell walls and roles of extracellular proteases in tumor progression are discussed in the review articles [9] and [10]. On the other hand, computational analysis, i.e., using bioinformatics tools, Ramirez et al elucidated the nature of interactions between the molecular chaperone OppA from Yersinia pseudotuberculosis and various protein ligands [11].

The role of enzymes was investigated by Alnoch et al. [12], who studied the immobilization and application of recombinant xylanase for the production of xylooligosaccharides used in the food industry. The effects of a mixture of tryptophan intestinal microbial metabolites on the activity of aryl hydrocarbon receptors, which play a key role in intestinal physiology and pathophysiology, were studied by Vrzalova et al. [13].

Compounds with anticancer potential were designed by Li et al. (cationic pillar[6]arene as inductors of cell apoptosis) [14] and by Gradova et al. (amphiphilic cationic chlorines for photodynamic therapy) [15]. Antimicrobial agents derived from natural compounds (salicylic acid and cinnamic acid) were studied by Pindjakova et al. [16] and Strharsky et al. [17]. Small molecules based on benzyl [4-(phenylcarbamoyl)phenyl]- carbamate scaffold, targeted as cholinesterase inhibitors for the treatment of Alzheimer’s disease, were designed by Kos et al. [18]. The biological activities of sophisticated and highly functionalized heterocyclic-based compounds were investigated by El-Kalyoubi et al. (5,6-diaminouracil-imidazolone derivatives) [19] and Empel et al. (pyridoquinothiazinium derivatives) [20]. Quinazolinones as antioxidant, cytotoxic, genotoxic, and DNA-protective agents were studied by Hricoviniova et al. [21]. The anti-inflammatory effect of T-2054 derivative of obeticholic acid for the suppression of osteoarthritis was described by Guo et al. [22], while *cis*-urocanic acid and its chelating properties were investigated by Bossak-Ahmad et al. [23]. Interesting and functional new modifications of the sex pheromone of the diatom *Seminavis robusta* were proposed by Bonneur et al. [24].

The application of spectroscopic techniques for the identification of superoxide dismutases was described by Kula-Maximenko et al. [25]. Masar et al. [26] focused on the evaluation of biologically active compounds using capillary electrophoresis, while the optimization of chromatographic techniques and conditions for the characterization of bioactive molecules, or for metabolomics and lipidomics, are discussed in the contributions of Szucs et al. [27] and Cajka et al. [28]. An interferometric light microscopy methodology for evaluating small cellular particles and liposomes was developed by Romolo et al. [29]. Physical and analytical measurements of the difference between solid–liquid interfacial adsorption of proteins in their native and amyloid forms were carried out by Abraham et al. [30]. Cryopreservation and its harmful effects on the endothelial integrity of human corneas were discussed by Rodriguez-Fernandez et al. [31], and the effect of UV light on photodegradation of acetylsalicylic acid was described by Daescu et al. [32]. An analysis of the stability of tetracycline hydrochloride in a hydrogel formulation and the overall anti-sebum activity was performed by Kostrzebska et al. [33].

As can be seen from the above, chemical biology as a progressive multidisciplinary interface not only enjoys great and respected attention from the world’s scientists and leading scientific teams, but is also widely supported by sponsors due to the expected significant achievements in the understanding of many diseases afflicting humanity in fast and effective solutions. Using a chemical biology platform, scientists use international research infrastructure (e.g., Instruct-ERIC [34]) to make cutting-edge technologies and methods used in structural biology available to each other, with the aim of driving and supporting innovation in the biomedical sciences.
